# Neural Circuits, Microtubule Processing, Brain’s Electromagnetic Field—Components of Self-Awareness

**DOI:** 10.3390/brainsci11080984

**Published:** 2021-07-25

**Authors:** Alicja Różyk-Myrta, Andrzej Brodziak, Małgorzata Muc-Wierzgoń

**Affiliations:** Faculty of Medical Sciences, University of Applied Sciences in Nysa, 48-300 Nysa, Poland; andrzej.brodziak@pwsz.nysa.pl (A.B.); malgorzata.muc-wierzgon@pwsz.nysa.pl (M.M.-W.)

**Keywords:** self-awareness, microtubule, electromagnetic field, mental imagery, ego dissolution

## Abstract

The known theories discussing the essence of consciousness have been recently updated. This prompts an attempt to integrate these explanations concerning several distinct components of the consciousness phenomenon such as the ego, and qualia perceptions. Therefore, it is useful to consider the latest publications on the ‘Orch OR’ and ‘cemi’ theories, which assume that quantum processing occurs in microtubules and that the brain’s endogenous electromagnetic field is important. The authors combine these explanations with their own theory describing the neural circuits realizing imagery. They try to present such an interdisciplinary, integrated theoretical model in a manner intuitively understandable to people with a typical medical education. In order to do this, they even refer to intuitively understandable metaphors. The authors maintain that an effective comprehension of consciousness is important for health care professionals because its disorders are frequent medical symptoms in emergencies, during general anesthesia and in the course of cognitive disorders in elderly people. The authors emphasize the current possibilities to verify these theses regarding the essence of consciousness thanks to the development of functional brain imaging methods—magnetoencephalography, transcranial magnetic stimulation—as well as clinical studies on the modification of perceptions and feelings by such techniques as mindfulness and the use of certain psychoactive substances, especially among people with self-awareness and identity disorders.

## 1. Introduction

Recently published articles enable improved cognizance of the essence of consciousness. Stuart Hameroff recalled the arguments in favor of the theory associating the nature of consciousness with quantum processing in the microtubules [1]. These arguments have recently also been discussed by Tianwen Li et al. [2]. Wider interest in this theory comes from the detection of high-frequency vibrations in the microtubules [3]. 

The progress in the realm of so called “field theories of consciousness” should be also noted. Recently, McFadden published in a trustworthy journal the convincing justification for his “conscious electromagnetic information field theory” [4].

Hence, it is now possible to relate these mentioned “Orch OR” and “cemi” theories to our explanation of how neural circuits realize imagery. We have presented the description of the recall of mental images from memory in several articles [5,6,7,8]. 

Making a brief review of the mentioned theories will allow us to endorse below a comprehensible theory integrating all these concepts, which will enable improved cognizance of the essence of the nature of self-awareness. 

It is necessary, however, to pay attention to a certain methodological difficulty. Contemporary theories explaining physiological processes are interdisciplinary and are based on fields of knowledge so advanced that they exceed the possibility to be fully understood by people with a typical medical education. We mean such areas as quantum physics and electromagnetic field theory. We believe that this difficulty can be overcome by proposing readers a presentation of the essence of such processes at a level that is understandable in an intuitive way, based on a glossary of basic concepts, developed based on reputable textbooks. We provide a glossary of the necessary terms as an Appendix A. We mark in this text, the subsequent references to such a glossary by specifying the number of a comment in parentheses, as {x}.

## 2. A Brief Description of “Integrate and Fire Neuron” Networks

Stewart Hameroff and Roger Penrose maintain that it is impossible to explain the phenomenon of consciousness based on the knowledge gained to date about the function of the network consisting of “integrate and fire” type neurons. Thus, there is a need for a brief reminder of the data related to such a network. 

All functional cells of the brain have some general characteristics. A neuron maintains an electric potential difference across the cell’s membrane. The negative charge of the interior is created by actively transporting sodium cations to the outside by an enzymatic mechanism, called the sodium pump. Stimuli reaching the nerve cell via synapses cause a decrease or sometimes an increase in the difference in the resting potential. If there are many such impulses in a relatively short time, the neuron’s excitation threshold will be exceeded. A significant potential difference will then be created in the vicinity of the hillock and then transferred as a wave along the axon and its branches. 

The recording of this change in potential at some place along the axon results in a sharp spike on the graph. This is designated as the action potential. Repeating the same stimuli hitting the receptors causes the frequent generation of action potentials, so-called firing, up to a maximum frequency of 500 Hz. 

When the action potential reaches the axon endings, arriving at the next synapses, it causes the release of a parcel of the neurotransmitter, which, by acting on the post-synaptic membrane, i.e., the wall of the subsequent neurons, changes their resting potential. Inducing changes in the resting potential, caused by stimuli arriving through synapses, existing on the dendrites of the neuron and over its entire surface is called spatial summation. The activity, the so called “weight” of each synapse, may be different. 

If the reactivity of the synapses of the middle segment of the neuron body (or the middle zone of the dendrites) lying in the first layer of the afferent pathways is high, and the activity of the peripheral synapses is low, then such a neuron will be sensitive to the macular image. Such neurons have already been detected in the retina by Stephen Kuffer [9]. The intuitively understandable illustrations of the neuron structure and function are available, for example, at: https://www.ncbi.nlm.nih.gov/books/NBK21535/ (accessed on 23 July 2021).

The experiments on cats performed by David Hubel and Torsten Wiesel showed that on the upper levels of the visual pathways, i.e., in the geniculate and striated cortex, neurons are sensitive to certain image patterns [10]. The higher the level, the more complex this pattern is. The different activity (“weights”) of the synapses on the dendrites and on the body of certain neurons makes it sensitive to a set of stimuli (“patterns”). A single neuron existing in a certain hierarchical structure is usually sensitive to several specific images. 

The brain handles sensory stimuli simultaneously in several afferent pathways of various modalities. The different aspects of the image falling on the receptors are perceived simultaneously. At the top of the hierarchical structures of any afferent pathway a group of superior, so called object neurons can be discerned. The existence of these object neurons was discovered by Gross and Mishkin [11,12]. It was proved later by Quiroga et al. [13,14]. 

The so called synaptic “weights” can be adjusted during the learning process [15,16]. 

**Figure 1:**
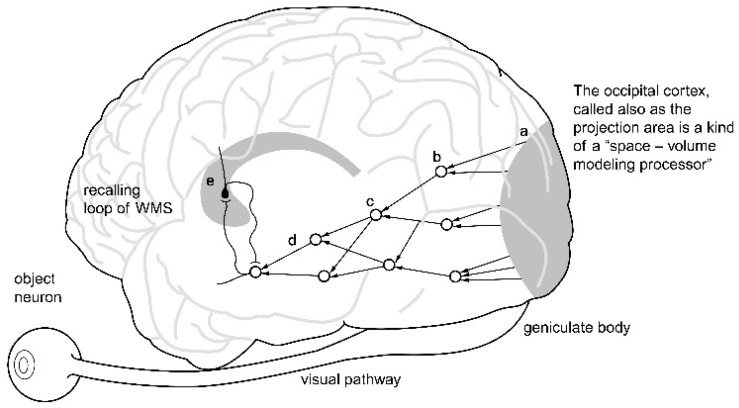
Diagram of the visual pathway, showing the location of object neurons and their relationship to memory systems. The diagram illustrates that the hierarchical neural structure, integrating visual information runs first to the occipital lobe, but is prolonged by afferent pathways in the temporal lobe, where so called ‘object neurons’ are located. The object neurons can be activated from the side of the speech area or by the loops of the working memory system.

Figure 1 illustrates the visual pathways which are anatomically folded. They run up to the occipital cortex. The stimuli are encoded here by neurons sensitive to patterns of line segments, inclined at certain angles. The processing of information is then performed by structures located in the temporal lobe of the brain. 

## 3. Theoretical Model of Neuronal Circuits Realizing Perceptions, Enabling the Memorization of Images and Their Recall from Memory in the Form of Imagery

The visual perceptions and the imagery of visually perceived objects are realized on the basis of the same neural substrates [5,6,7,8]. This fact has been confirmed recently by several researchers [17,18,19].

Joel Pearson and his coworkers put it in words “that visual mental imagery is a depictive internal representation that functions like a weak form of perception” [17]. 

Nadine Dijkstra with coworkers develops this idea and writes that: “For decades, the extent to which visual imagery relies on the same neural mechanisms as visual perception has been a topic of debate” [18]. These researches review recent neuroimaging studies and conclude that “there is a large overlap in neural processing during perception and imagery” and that “neural representations of imagined and perceived stimuli are similar in the visual, parietal, and frontal cortex” and that “perception and imagery seem to rely on similar top-down connectivity” [18]. 

Rebecca Keogh and coworkers emphasize that: “Visual imagery—the ability to ‘see with the mind’s eye’—is ubiquitous in daily life for many people; however, the strength and vividness with which people are able to imagine varies substantially from one individual to another” [19]. These researchers present the results of brain imaging and transcranial magnetic phosphene data and conclude that “lower resting activity and excitability levels in early visual cortex (V1–V3) predict stronger sensory imagery” [19]. 

**Figure 2:**
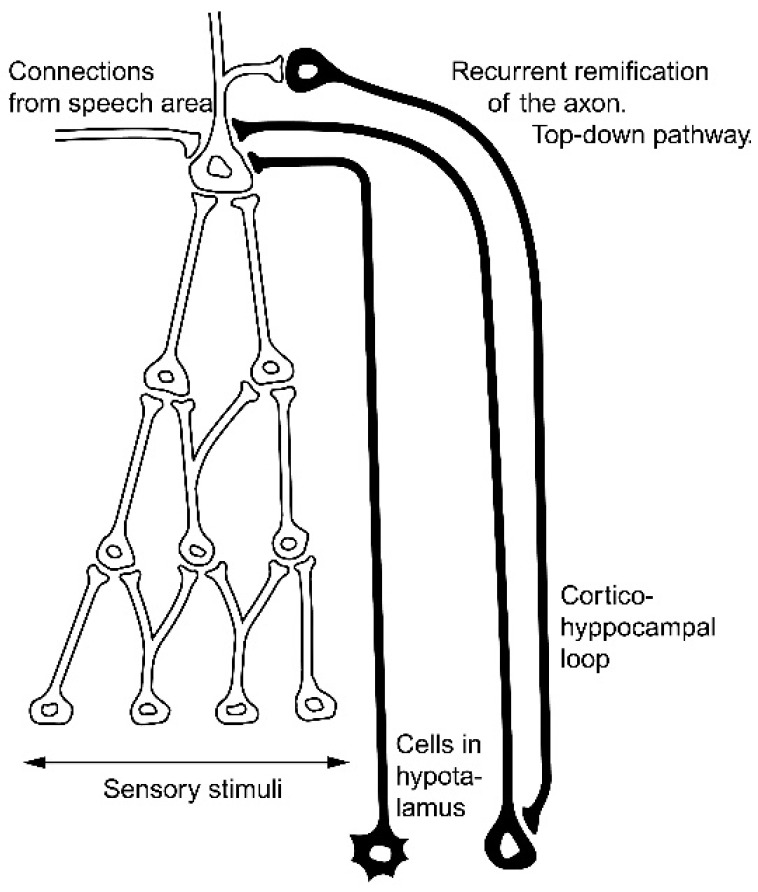
There are three types of connections with the object neurons. Considering the neuronal structure that is active during the perception of a certain object, the connections from the side of the hypothalamus and amygdale, which are active at the time of learning, should be distinguished from connections from the side of the hippocampus where are the elements of the working memory system. Excitation of the object neuron from the side of the speech area is sustained by the cortico-hippocampal loops of the working memory system.

**Figure 3:**
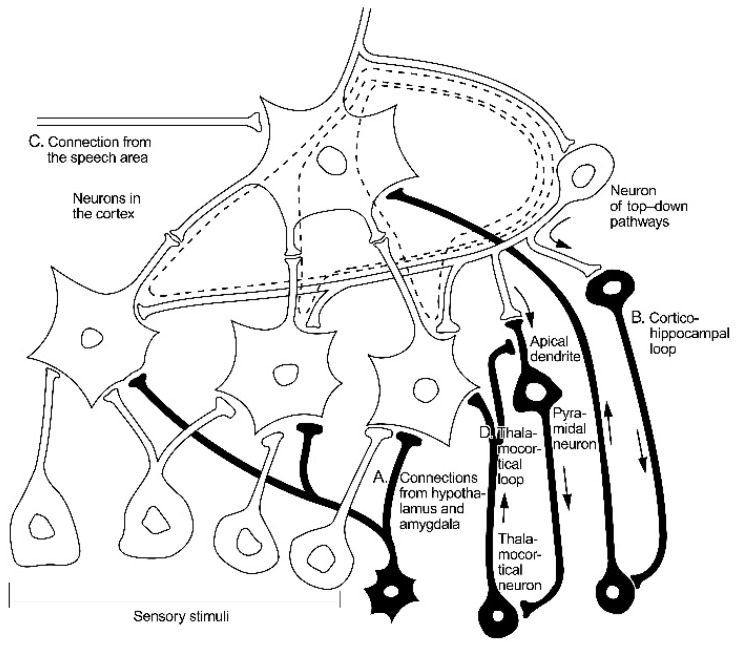
Diagram of the circuits responsible for perceptions and recalling of mental images. Diagram of hierarchical structure of neurons integrating sensory information. The long-term memory is consolidated under the influence of connections from the hypothalamus and amygdale (A). The cortico-hippocampal loops (B) are elements of the working memory system recalling mental images. The activation of the object neuron by the cortico-hippocampal loop induces recurrent reactivation of neurons in lower layers. The dotted line indicates the circuits of repetitive propagation of stimuli in the upper layers of the hierarchical structure, which is the essence of imaginary. The activation of an object neuron can also be evoked from the side of the speech area (C). The thalamo-cortical loop (D) reach the lower parts of the cortex. The oscillations in these circuits happens e.g., in periods of a deep sleep. The element “D” of the diagram emphasize that the brain’s actions are based on an intrinsically generated neuronal activity and that the sensory inputs are acting as modifiers of such intrinsic activity. The figure facilitates the understanding of why perceptions and imagery stimulate further oscillations in the upper layers of the hierarchical structure.

The structures essential for the realization of imagery are illustrated in Figure 2 and Figure 3.

These figures are reproduced from our previous papers presenting a theoretical model of neural circuits, which explains how three distinct basic cognitive functions are realized [5,6,7,8]. In formulating this model, we assumed that it is necessary to explain the difference between the perception of novel and unfamiliar objects. This model also explains the process that takes place when we imagine familiar objects in the form of mental images. The simplest example of evoking mental images is stimulation from the speech area, which remembers the verbal descriptions (names) of familiar objects. Referring briefly to this theoretical model, we should become familiar with some basic data about the neuronal substrate of these processes. 

Cortical neurons have recurrent axons, which activate interneurons going to the lower levels. When excitation reaches the object neurons at subsequent moments, neurons from lower levels are stimulated secondarily. Thus, there are conditions for the circulation of impulses between neurons of higher and lower levels of afferent pathways. It has been experimentally confirmed that such oscillations occur [20,21,22,23,24].

Thus, if object neurons are excited from the side of the speech area, there is a circulation of impulses between neurons of the higher and lower layers of the same hierarchical structure that was excited during the perceptions of this object. These oscillations are the physical substrate of imagination. During complex mental processes like searching for solutions, the working memory structures instantiate the necessary, useful mental images [8]. This maintenance of imaginal activity is accomplished by the cortico-hippocampal indexing loops. 

When we repeatedly perceive an image, the synaptic weights of the hierarchical structure active during such perceptions is altered and a learning process takes place as we remember the pattern of the learned object. If a familiar object is perceived, there is secondary activity of the cortico-hippocampal indexing loops; in this way, the structure of the known, recognized object is stimulated from two directions. 

## 4. Review of Stewart Hameroff’s and Roger Penrose’s ‘Orch OR’ Theory

Penrose and Hameroff proposed their theory because they remarked that evidence related to the “integrate and fire” network of neurons does not indicate how consciousness could arise from its activity [25,26,27,28,29]. 

Their theory was inspired by the observation that protozoa such as Physarum exhibit intelligent behavior; that is, they can learn, remember, and solve problems such as escaping from a maze, even though their organisms do not contain synaptic connections. Nonetheless, these protozoa have a cytoskeleton. Stuart Hameroff remarked that neurons also have an organized cytoskeleton, composed of microtubules and persuaded Roger Penrose to collaborate in the formulation of a theory linking quantum physics with brain function [25,28,29]. 

The researchers thoroughly analyzed the structure of microtubules [25]. They found in their structure elements that may play a role similar to the components of quantum information processing systems. Microtubules are composed of 13 protein filaments, each of which is a series of tubulin dimers. Tubulin is a kind of globular protein. Microtubules are assembled from dimers of α- and β-tubulin. In the cell bodies of neurons, the microtubules are arranged in short scattered segments connected by microtubule-associated proteins (MAPs). Microtubules in axons are arranged in continuous, long bands.

The tubulin dimers have pockets that contain delocalized electrons {1,2}. The authors assume that the electrons of the mentioned pockets could represent quantum states and become quantum superposed {3,4}. Therefore, they claim that these electrons represent coherent quantum superpositions, in other words, so called qubits {5}. They also assume that these electrons can be quantum entangled {6}. 

**Figure 4:**
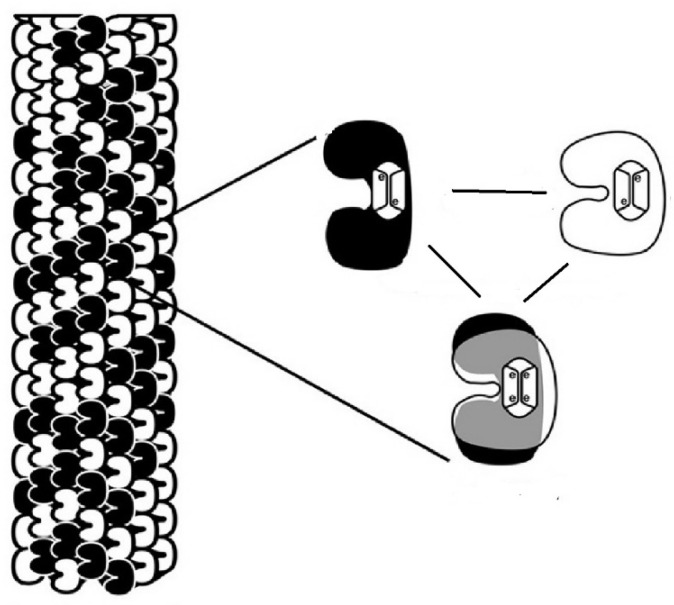
Symbolic presentation of a microtubule composed of tubulin dimers existing in three different states. Symbolic presentation of tubulin dimers, which due to the positions of delocalized electrons can be in two different states and additionally in a quantum superposition state. On the left a symbolic presentation of a microtubule composed of tubulin dimers existing in three different states.

**Figure 5:**
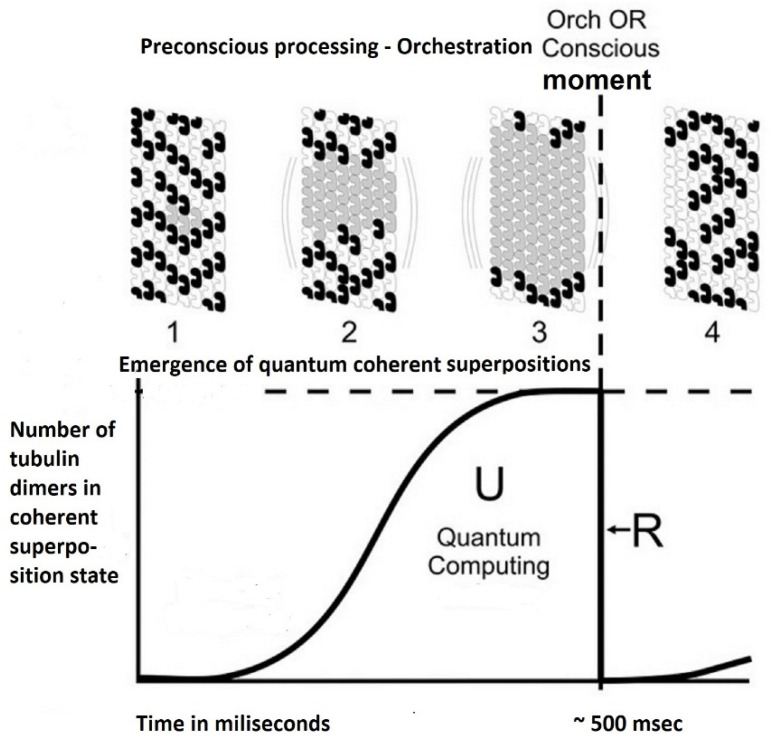
Symbolic illustration of the essence of Roger Penrose and Stuart Harmeroff’s ‘Orch OR’ theory. The authors assume that in the microtubules of neurons repetitive, cyclic quantum information processing is realized, which consists in increasing the quantum coherence of the tubulin dimers, interrupted by the OR operation, causing the conscious moment. In larger synchronized sets of neurons, the termination of orchestration by OR moment occurs after approx. 300 to 500 ms.

In one of their first papers related to the ‘Orch OR’ theory, the authors presented intuitively understandable drawings [25]. We present here in Figure 4 and Figure 5 a simplified modification of these drawings.

The authors of the theory believe that the basic process establishing consciousness is cycles of increasing synchronization, as they say ‘orchestration’ (Orch), which they equate with achieving quantum coherence {7} and subsequent moments of decoherence {8}, denoted as the OR operation [30,31,32,33].

Considering the processes occurring simultaneously in many neurons of the afferent pathways, signals coming through the synapses stimulate the orchestration process and when the threshold in a particular neuron is reached, so called objective reduction (OR) occurs.

This is equivalent to making a choice or decision. The obtained state is then transmitted to the axon hillock region. The quantum processing in dendritic and somatic microtubules determine axonal firings, which determines conscious behavior [33].

The researchers also claim that singular quantum effects, by reason of bonding coherence, are magnified and influence the function of the brain on a large scale [33].

An interesting consequence of the theory is that consciousness occurs on the basis of discrete events called “conscious moments”, which happen at a frequency of about 24–90 Hz. Thus, consciousness emerges as result of series of discrete events like the sequential frames of a movie. This synchronization happens in particular in the parietal and frontal lobes of the brain. 

It is generally known that the activity of the neural network is synchronized. The registration of EEG waves in the alfa, gamma, delta range is a manifestation of this synchronization. Large amounts of data have also already been collected on the synchronous operation of neurons in different, sometimes distant centers of the brain, but active in the course of specific, distinguished activities. It is assessed by measurements of so-called connectivity [34,35,36,37,38]. The authors of the theory consider the phenomena, which could be responsible for this synchronization. They emphasize the role of gap junctions, which exist between adjacent neurons. They assume that quantum states in the microtubules of a neuron can be enhanced by entanglements and tunneling through the gap junctions of adjacent neurons. They cite evidence that gamma waves, the known correlate of consciousness, rely on gap junctions. They also assume that spatially separated microtubules of different neurons can be quantum entangled. Thus, Hameroff and Penrose ground the process of the emergence of consciousness not only in the quantum processing of data in microtubules, but also in achieving synchronization of neuronal activity in vast stretches of the brain. 

People becoming acquainted with the ‘Orch OR’ theory face the same difficulty as readers studying works on quantum physics. It is known that physicists continue disputes regarding the problem of the so-called reduction of the wave function {9}. It should be noted that Stuart Hameroff and Roger Penrose propose some personal beliefs in the frame of the “Orch OR” theory.

They tried to replace the term “acquiring coherence” with the words “achieving orchestration”. Roger Penrose also proposed his own concept of wave function reduction, which he termed “objective reduction” (OR). He claims that ‘OR’ occurs spontaneously “due to an objective threshold in the fine-scale structure of the universe at time *t* = ħ/EG, where ħ is the Planck-Dirac constant, and EG the gravitational self-energy of the superposition” [33].

It is a considerable intellectual challenge for readers of Hameroff’s and Penrose’s papers to realize what the authors mean by “gravitational self-energy of the superposition”. 

As an introduction to the attempt to understand this concept, it should be recalled that different types of radiation, e.g., gamma radiation, beta radiation, and the corpuscles corresponding to them, including photons, are assigned different energy levels. Physics describes the behavior of various types of radiation and particles in a gravitational field. The distinguished “gravitational self-energies of the superposition states” are extremely small energies. Notwithstanding, it should be noted that if we consider these energies not for a single pair of superimposed quantum states, but for a larger system, then the gravitational self-energy is calculated for the whole such set of elements. The time after which the mentioned “objective threshold” is exceeded followed by the OR operation is thus different and depends on the extent of the entangled quantum states. As already mentioned, this time for the human nervous systems is in the order of 15–300 ms. 

The authors also assume that “at each such OR moment random (proto-) conscious moments of experience occur, composed of basic ‘qualia’” [1,33]. They remember the meaning of this notion saying that “qualia yield, the taste of chocolate, the smell of lilac, the touch of soft skin or the feeling of love” [33]. 

Stuart Hameroff and Roger Penrose often refer to the notion of quantum information processing or quantum computing realized in the microtubules of neurons {10}. However, what is interesting is that they raise the problem that the “understanding” of ‘Orch Or’ processing “cannot be explained by any computational system and must derive from some ‘non-computable’ effect” [26,33,39].

The supporters of the ‘Orch OR’ theory highlight new experimental data established in recent years regarding the discovered properties of microtubules. Satyajit Sahu, Subrata Ghosh, and Anirban Bandyopadhyay proved that microtubules can exhibit resonant oscillation that causes the vibrations of axons and that a single extracted microtubule is a memory-switching element [3,40]. They maintain that the properties of the microtubules are similar to a random-access memory, analogous to a flash memory switch used in computer chips. 

The enormous capacity of information processing in microtubules is probably used not only to determine the actual activity of the neuron axon undergoing firing. Most likely, non-computable quantum processing in microtubules is necessary for the experience of qualia. 

It is known that manifestations of macroscopic quantum effects such as superconductivity and superfluidity are observed in systems operating at low temperatures. The current, practical possibilities of implementing quantum computing are obtained in systems operating at low temperatures. Therefore, the authors of the theory also address the problem of how quantum data processing can take place in human organs, and in human brains. In their articles, this dilemma is formulated by asking how it is possible in an environment, which is “warm, wet and noisy” [33,41]. They try to dispel these doubts by pointing to the already known numerous examples of functional quantum effects in living organisms. As arguments, they recall data on the quantum mechanisms occurring during photosynthesis, the perception of smells, and the navigation of bird flights. The authors are convinced that microtubules have the property of isolating quantum information processing, which is sufficient to obtain the necessary coherence of these systems, at least, in short time intervals, necessary to realize so-called conscious moments.

Readers with a background in biology and medicine need some kind of intuitive approximation for these considerations. We suggest using for this purpose the terms presented in the attached glossary. It is important, however, to reach an approximative understanding because the discussed theory is useful to explain known conscious sensations. 

The considered theory facilitates comprehension of the occurrence of the “feeling of qualia”. In another words, the theory explains how a “bright, colorful, panoramic, sometimes sound replica of the outside world” is built in our minds. 

Many animal species probably develop feelings known as qualia. Nevertheless, it has long been known that only some of them recognize themselves in a mirror [42,43]. Thus, the need arises to explain what the process of the perception of one’s own identity is about; that is, what self-awareness is.

## 5. Overview of the Brain’s Endogenous Electromagnetic Field Theories

Among many theories explaining the essence of consciousness, the so-called field theories of consciousness should be distinguished. According to all these concepts, consciousness is considered an essence which also has an extension in space. The authors of the majority of these theories assume that consciousness is grounded in particular features of a physical field. At least one of these theories proposes that consciousness is identical to a hypothetical non-physical field; nonetheless, which also has an extension in space. Such a point of view was proposed by Benjamin Libet, who formulated the theory of the cerebral mental field (CMF) [44]. 

There is growing recognition of the theories of consciousness related to an electromagnetic field [45,46,47,48,49,50]. Recently Johnjoe McFadden published in a trustworthy journal the convincing justification for his theory, developed over twenty years, which he denotes as “the conscious electromagnetic information field theory (cemi)” [4]. 

Readers wishing to familiarize themselves with the “cemi” theory should recall elementary data related to the generation of a magnetic field around a conductor carrying direct or alternating electric current {11}. The most important elementary fact is that an electromagnetic field arises around a conductor carrying an electric current, which occurs only when there is a closed circuit. Nonetheless, the basic data on neural networks emphasizes data on the transmissions of waves of excitation (action potentials) occurring along the neural axons, i.e., they consider the transmission between the neurons of lower and higher levels of, e.g., afferents pathways. An endogenous magnetic field is created within the brain, which is recorded during magnetoencephalography. Thus, there are closed electrical circuits in brain tissue. They are considered when we take into account the pathways running backwards to the lower levels. 

The authors of theories of consciousness related to an electromagnetic field assume that the basic process evoking this phenomenon should consist not only in actions occurring over time but also causing the formation of a certain spatial wholeness [4]. Johnjoe McFadden points out that neural networks realize step by step time-progressive process. He argues that when looking for a physical medium supporting something that has the feature of a spatial structure, one must appeal to physical fields [4]. Therefore, McFadden remarks that an electromagnetic field can integrate information in space. He concludes that “consciousness is information physically integrated, and causally active, encoded in the brain’s global electromagnetic (EM) field” [4].

McFadden also remarks that propagating neuronal potential changes (firing, action potentials) induce the formation of magnetic fields which overlap and combine to generate “the brain’s global EM field” [4]. He remarks that the human brain can be conceived as the assembly of “around 100 billion EMF transmitters” [4]. The electric phenomena are routinely recorded by electroencephalography (EEG) and the EM field is also routinely detected, assessed and recorded by magnetoencephalography (MEG).

The author emphasizes, though, that consciousness occurs when there is a massive synchronization of neuronal activity and also when repetitive oscillations in neuronal circuits occur. He emphasizes that conscious neuronal processing should be associated with “re-entrant circuits, essentially closed loops of neuronal activity whereby neuronal outputs are fed back into input neurons” [4]. 

Since the aim is to provide readers with an explanation of the latest theories of the essence of consciousness in a way that can be comprehended intuitively, we will again use metaphors. The postulate of the role of the electromagnetic field of the brain can be illustrated with an analogy to people participating in a concert, performed by a symphony orchestra. In this situation, known from personal experiences, we can discern analogical elements. A field is also used here, in this case, it is an acoustic field, which enables the propagation of acoustic waves. The musicians in the orchestra must synchronize their actions in order to obtain the desired effect. In this situation of synchronous work, the sounds generated by many musicians, the members of the orchestra, create a certain wholeness (entity) in the acoustic field, perceived by the audience. This entity created in the space of the concert hall by means of the acoustic field changes over time so that we perceive the melody. In this analogy, it is clear that the effect, for example, of perception of the ‘Imperial Waltz by Johann Strauss’, would not be obtained, if within the range of the acoustic field, existing in the concert hall, a certain ‘wholeness’, composed from single sounds emanating from the members of the orchestra, was not performed in a synchronous way. 

It seems to us that the efforts to describe the structure and function of the electromagnetic field of the human brain are at the very beginning of possible development. It should be noted that electromagnetic phenomena are composed of tiny elements. Colin Hales and Susan Pockett formulated the description how so called ‘local field potentials’ (LFPs) participate in the creation of the brain’s electromagnetic field [50]. They write: “the primary need is to attend to the genesis of the electric and magnetic fields of the brain at the level of tissue ultra-structure, via spatiotemporally coherent systems of source charge density and source current density centered on the neural membrane” [50]. 

Neuroscientists who will improve the description of the structure and function of the brain’s electromagnetic field will probably also take into account the phenomena of induction {12} and interference {13}. It is possible to formulate a hypothesis that the circulation of impulses in the neuronal circuits of subcortical structures can evoke by induction some components of the magnetic field in the upper regions of the brain. It is possible that induced phenomena are involved in the still implicit ability of the brain structures to act synchronously [51]. 

McFadden additionally emphasizes the key “neural signatures of consciousness”. He remarks that the feeling of self-awareness is conditioned by repetitive oscillations in neuronal circuits of the parietal and prefrontal areas of the brain, as well as synchronized long distance connections in the whole brain [4].

## 6. The Theoretical Model Integrating the Most Convincing Explanations of the Phenomenon of Consciousness

We believe that the attempts to explain the nature of consciousness discussed above can be integrated and presented in the form of a theoretical model that takes into account several components of this phenomenon.

Contemporary neural science describes in detail the operation of neural circuits active during perception. The available functional brain imaging techniques have also made it possible to determine the importance of various brain centers in the execution of particular activities. Their numerous connections and cooperation have also been established. An important neurophysiological feature is the synchronicity of their actions, which is assessed by so called degrees of connectivity [34,35,36,37,38].

For a long time, scientists have emphasized the need to explain the essence of fundamental sensations such as colors, tastes, sensory perceptions, which is referred to as the “feeling of qualia”. The claim of Roger Penrose and Stuart Hameroff that their ‘Orch OR’ theory effectively defines the theoretical model of these feelings seems to be convincing.

There are, though, other cognitive processes than perceptions. For example, when we search for a solution to a situation, imagination is indispensable. In our former papers we described neural circuits, which are necessary to recall mental images, in other words, to realize imagery. This theory is illustrated briefly in Figure 3. Our concepts of neural networks realizing imagery have been quoted by many neuroscientists [52,53].

It is known, however, that only some primate species have the capability of self-recognition [42,43]. Thus, it should be possible to indicate the neuronal structures responsible for the feeling of identity, i.e., for a representation of the subject, or in other words, for the capability to be aware of one’s self. To put it another way, one should be able to explain what the meaning is of the word “I” or considering it in the light of the psychoanalytical theories, what is denoted by the term “ego”. Some indications come from introspection.

The feeling of identity, also the understanding of the word “I” is based on autobiographical memory [54]. The comprehension of the word “I” requires to recall “who I am”. This, nonetheless, is established by the memorized biography. 

The proposal of the nature of the feeling of one’s self can be derived from our theoretical model of imagery. We described a structure forming the mental image of a certain object. Probably the feeling of one’s self appears when there is a perception of one’s own body and environment. In order to accurately imagine yourself, it is additionally necessary to imagine one’s past and recall images of one’s environment, which also allow a person to imagine his foreseeable future. 

On the other hand, Rodolfo Llinas and Georg Northoff convinced most contemporary neuroscientists that one of the basic mechanisms of consciousness consists in the self-excitation of a neuronal network composed of thalamo-cortical pathways. They describe this activity as re-entrant processing [55,56]. Probably the existence of neural circuits which trigger themselves is a very basic rule of nervous systems. 

McFadden remarked, however, that the key “signature of consciousness” is re-entrant processing in the parietal and frontal areas [4]. 

These statements are in agreement with known findings that impairment in the prefrontal and parietal regions lead to deterioration of self-awareness of persons suffering from Alzheimer’s disease and identity disorders [57]. Michael Kopelman, considering disturbances in the retrieval of autobiographical memory, emphasizes the importance of frontal ‘control’ systems in interaction with medial temporal and hippocampal systems [58]. The contribution of prefrontal executive processes to creating a sense of self is recognized by many researchers [57,58,59,60]. Researchers who successfully visualized the change in brain function after administering psilocybin make reference to psychological terms when discussing self-awareness disorders, talking about “ego-dissolution” [61].

It seems to us that the integrated theory of self-awareness should take into account one more important phenomenon, namely the necessity to focus attention on a specific area of considerations at every moment of the conscious state. 

The introspective experience shows that if our thoughts or, in other words, our working memory activity are about solving a difficult problem, we will not be able, for example, drive a car safely. Our attention (stream of consciousness) can be concentrated efficiently only on one issue (one operational task). It seems that such focusing of attention, known from psychological introspection, on the neuronal level, manifests itself in a momentary synchronization of the activity of specific neural circuits.

This is consistent with the general rule that if distant brain centers participate in the performance of a certain mental operation, then the activity of the neurons of these centers is synchronized for the duration of this operation.

It is important that among the literature of neuroscience we find statements about this important component of self-awareness, which is the ‘focusing of attention’. These statements relate to intermittent synchronization in the gamma band.

Lyall Thompson writes: “Working memory (WM)—the ability to keep information in mind for short periods of time—is linked to attention and inhibitors abilities, i.e., the capacity to ignore task-irrelevant information. These abilities have been associated with brain oscillations, especially parietal gamma and alpha bands…” and below: “we concluded that parietal gamma oscillations, therefore, modulate working memory recall processes…” [62].

Supratim Ray and John Maunsell are of a similar opinion as they write that: “Gamma rhythm (which has a center frequency between 30 and 80 Hz) is modulated by cognitive mechanisms such as attention and memory, and has been hypothesized to play a role in mediating these processes by supporting communication channels between cortical areas or encoding information in its phase…” [63].

Other authors emphasize that synchronization in the gamma range takes place also during the consolidation of memory traces [64,65]. Drew Headley and Denis Paré write: “Regions known to participate in the formation of emotional memories, such as the basolateral amygdala, also promote gamma-band activation throughout cortical and subcortical circuits. Recent studies have demonstrated that gamma oscillations are enhanced during emotional situations…” [64]. 

We should take into account the remark of György Buzsáki and Xiao-Jing Wang who précised in their extensive review paper that: “gamma oscillations are short-lived and typically emerge from the coordinated interaction of excitation and inhibition, which can be detected as local field potentials” [66]. This statement means that the occurrence of momentary synchronization in the gamma band in a certain group of neurons can only start or close a certain mental operation.

The exemplary synchronization of the activity of a certain group of neurons in the course of performing a specific mental operation is characteristic for many different cognitive processes [67,68].

Siying Xie and coworkers discuss, for instance, an important phenomenon that during visual imagery and perception occur the synchronization in the Alpha Frequency Band [67]. 

Gregor Leicht and coworkers examined the gamma-band synchronisation in a frontotemporal auditory information processing network [68]. 

In trying to integrate the presented data, we can say that among “neural signatures of consciousness” there are manifestations of several levels of re-entrant processing. One should also note that when consciousness emerges, an electromagnetic field of a complex structure is created and when self-awareness appears, re-entrant processing in parietal and prefrontal circuits takes place. The activation of centers in the parietal and prefrontal regions is probably necessary for self-imagining. Perhaps this is the moment when the subject’s perception of the world “I” is felt. Probably the realization of the self-image (the image of oneself) is an indispensable condition for the feeling of self-awareness. 

Presenting our attempt to integrate several theories, aimed at a better understanding of the essence of self-awareness, we should also respond to new, recent observations regarding the importance of data processing in the prefrontal regions of the brain. 

Braam van Vugt’s research shows that the prefrontal cortex is one of the brain regions that mediates visual consciousness [69]. Moreover, Madhura Joglekar provided evidence that the prefrontal cortex is important for igniting neural networks that contribute to visual signal processing [70]. 

These observations may seem paradoxical, as we all associate the pre-processing of visual data mainly with the distant occipital lobes. 

The discussion of the interaction of several elements necessary to have a sense of self-awareness, presented in this article allows probably to comprehend better why the prefrontal cortex is involved in processing of visual data. 

It should be remembered that in order to be self-aware one need, among others: to experience embodiment, i.e., the so-called bodily self-consciousness, take into account feelings associated with programming and performing movements and complex activities with observation of their consequences, integrate data about one’s past (autobiographical memory) and even of an image of the outside world and remembered opinions about oneself. Moreover, the experience of self-awareness requires performing mental operations to put oneself in the perspective of the others. The data processing operations listed above take place in different regions of the brain. No wonder then that a certain derivative of visual experiences is also necessary at the highest level of integrating data about oneself, which occurs in the prefrontal cortex. 

It is a popular simplification, a kind of metaphor, but nonetheless a useful mental shortcut if we say that in the prefrontal lobes there is an essential part of the “I”, the subject, the “homunculus” that looks. This metaphor will seem less bizarre if we consider what we know about “the neural correlates of dreaming”.

“Francesca Siclari with collegues, already in the title of their paper state that they established the neural correlates of dreaming [71]. Their article is cited by many researchers. However, it is known that Perrine Marie Rub in a polemical article states: “… no reliable (neuro) physiological correlates of dreaming have been identified yet which means that one cannot know whether a sleeper is dreaming or not while she/he is sleeping” and that: “Our only access to dreaming is still dream reports, which are made a posteriori, during wake and which are possibly partial and/or modified by the waking consciousness” [72]. 

Perrine Marie Rub explains that although there are no precise data on regions active during dreams, it has been established, which centers are active after waking up, when people often and efficiently recall and report data on the content of dreams. This author states that “It is known for a long time now that a lesion… in the temporo-parietal junction is associated with a cessation of dream reports [72].

Serena Scarpelli states, however, that: “… several studies confirmed that the posterior parietal area and prefrontal cortex are responsible for dream experience” [73].

It seems to us, that in the context of the presented theories of consciousness, on the basis of phenomenological considerations, the following remarks can be made however: We can deliberate if the person who has vivid dreams is conscious? and if not, what is the difference between the dreaming state and the waking state. 

It seems to us that emphasizing the difference will be precise if we introduce the category of the so-called “stories”. It should be noted that people who are awake perceive the sequence of events that occur, which consists on changes in the environment, new situations and possible own actions. The content of the processed data is mainly perceptions, less imagery. A conscious person has a sense of having control, at least partial control, over the events that occur. Moreover, a conscious person may pause his own actions and formulate “a story about what happened”. A conscious person also has control over his actions and over what he focuses for his attention on.

A person in a dream state, sense the experiences similar to imagery and has no control over the development of the transformations of subsequent experienced images. A person in the course of such a dream not only cannot act according to his own will, but is also unable to focus attention on selected elements of the landscape being experienced. A dreaming person has the impression that someone else is the scriptwriter of the experienced impressions. Only after waking up is it possible to try to consciously formulate a “story about what occurred during the dream”.

So, a question arises, that has been bothering people for a long time, namely who the author of the dream sequence scenario is. A provisional answer to this question comes down to the hypothesis that “this author” it is a neural representation of “personage”, which is in part constituted by the above-discussed structures responsible for comprehension of the word “I”. It should be noted, however, that while we are awake these structures consist, as we wrote above, of self-perception and self-image (imagination of oneself). During sleep, in the absence of sensory perceptions, only the part of this structure that results from “self-image” is active. So it can be said metaphorically, that this is a different “personage”, moreover, who is less embedded in the realities of the world.

Nowadays, changes in the experience of self-consciousness can also be studied through the use of so-called transcranial magnetic stimulation (TMS). The discharge of impulses of transcranial magnetic stimulation directed at the prefrontal area and parietal regions is effective in treating depression and borderline personality disorders [74]. The applications of TMS interfere with the electromagnetic field of the brain, especially with the so called ‘default mode network’ (DMN). The use of transcranial magnetic stimulation also helps to realize that the generated electromagnetic field has a secondary, as it were, feedback influence on the activity of neuronal circuits. This is described in detail by Colin Hales [48].

The advancement of magnetoencephalography and of transcranial magnetic stimulation will empower fast progress in the understanding of the features of the brain’s endogenous electromagnetic field. 

Thus, the integration of the mentioned theories facilitates a conclusive answer to the questions about the nature of self-awareness. Only multi-level hierarchical data processing, embracing the areas placed in the parietal and prefrontal lobes makes it possible to pass the mirror test successfully.

It appears that a persuasive explanation of the phenomenon requires, apart from an indication of neural substrates, also a description of the role of a spatial field, namely of an electromagnetic field, which carries energy and has a particular shape.

Moreover, the attempt to explain what self-awareness is leads to the conclusion that first it is indispensable to explain how the images are realized. Next, it involves the necessity for an explanation of what the ‘image of oneself’ is. Then, having already a model of the ‘mental image of oneself ‘, we indicate a particular, specific process. This particularity can be explained if we move to the meta-level of inference. 

This transition is analogous to describing first the perception of a certain geometric figure, for example, a triangle, a parallelepiped (rhomboid) and then to describing how to imagine a rotation of the parallelepiped. The gathered knowledge about the neural substrate and operation of a mental rotation is an effective example of an extended form of imagery [75,76,77,78]. Marc Jeannerod emphasizes that self-identification also relies on the congruence of self-generated movements and their expected consequences [79]. Hence, there are arguments indicating that self-awareness is based on sophisticated forms of imagery. Such extended forms of imagery involve a mental operation on objects from the world of Platonic ideas. Thus, it is highly probable that Roger Penrose was right when discussing the essence of consciousness; he postulated the necessary connections of the mental world not only with the physical world but also with the world of Platonic ideas [26]. 

## Data Availability

Not applicable.

## Appendix A

A glossary of elementary concepts in quantum physics and electromagnetic theory.

{1} Delocalized electrons are not associated with a single atom or a covalent bond. In chemistry, this notion refers to resonance in aromatic molecule compounds. In chemistry, this notion refers to delocalized electron density shared between all the atoms in the ring of an aromatic molecule compound. In solid-state physics, this refers to free electrons that facilitate electrical conduction. In quantum chemistry, this refers to molecular orbital electrons that have extended over several adjacent atoms.
{2} Atomic orbital. In atomic theory and quantum mechanics, an atomic orbital is a mathematical function describing the location and wave-like behavior of an electron attached to an atom. This function can be used to calculate the probability of finding any electron of an atom in any specific region around the atom’s nucleus. The visualization of the concept of an orbital is difficult. The orbital can be compared to a three-dimensional, long-exposure photograph of an electron’s motion around the nucleus. Such a photo would show the area where the electron moves.
{3} Quantum superposition it is a concept of quantum mechanics. This principle states that any two, or more quantum states can be joined together, as it is said “superposed” and the result will be another quantum state. Every quantum state can be represented as a sum of other distinct states.
{4} The principle of quantum superposition states that a composed quantum state is a combination of all possible states, where the contribution of each configuration is specified by a complex number.
{5} A qubit is a two-state quantum system, like the spin of the electron, where two levels as spin up and spin down can be considered or the polarization of a single photon where two states can be taken to be the vertical polarization and the horizontal polarization. In a classical information processing system, a bit would have to be in one state or the other. Quantum mechanics allows the qubit to be in a coherent superposition of both states simultaneously. When qubits are measured, the result is always either 0 or 1, but the probabilities of these two outcomes depend on the quantum state that the qubits were in immediately prior to the measurement. This property is fundamental to quantum mechanics and quantum information processing or quantum computing.
{6} Quantum entanglement is a phenomenon that occurs when a pair or group of particles cannot be specified independently of the state of other elements, even when the particles are separated by a large distance. In entanglement, one component cannot be fully described without considering the other. The state of an entangled system is always defined as a superposition of constituents. An important difference between qubits and classical bits is that some qubits can be in quantum entanglement.
{7} Coherence and quantum coherence. Coherence describes the properties of the physical quantities of waves. The coherence of two waves happens when their frequency and phase difference are identical and constant. Quantum coherence. Quantum physics concern an object which has wave-like properties. Electrons are perceived here as waves. Particles such as electrons are described in quantum physics by a wave function, a mathematical description of their quantum state. When a definite stable phase relation exists between different states, the system is considered as coherent. A precise phase relationship is necessary to perform quantum computing on information encoded in quantum states. When quantum systems are isolated, they can maintain coherence, but it is impossible to manipulate or investigate them. If a quantum system is not perfectly isolated, for example during a measurement, coherence is shared with the environment and is lost within time in a process called quantum decoherence.
{8} Quantum decoherence can be viewed as the loss of features described above as characteristics of coherent systems. During decoherence, information maintained by a quantum system is lost into the environment. It has the effect of sharing quantum information with the surroundings.
{9} The “collapse of the wave function” known also as the “reduction of the wave function”. In quantum mechanics, wave function collapse occurs when a wave function of several superposed quantum states reduces to a single concrete state due to interaction with the external world. This interaction is sometimes called an “observation” or “measurement”. The collapse of the wave function happens by reason of decoherence. The components of the wave function are then decoupled and acquire phases determined by their immediate surroundings.
{10} Quantum computing is based on quantum phenomena such as superposition and entanglement. Systems performing computations using these phenomena are known as quantum computers. There are several kinds of quantum computing systems like the quantum circuit model and the quantum Turing machine. The most widely used model is the quantum circuit. A quantum circuit is a network of quantum gates connected by wires. Quantum gates represent quantum operations, while the wires represent the qubits on which the gates act. A significant technological requirement for quantum computers is the ability to maintain coherence at room temperature conditions.
{11} Macroscopic scale quantum coherence enables novel quantum phenomena, like the action of a laser, superconductivity and superfluidity. The Bose–Einstein condensate, existing in temperatures very close to absolute zero (−273.15 °C) is also an example of a system displaying macroscopic quantum coherence. The classical electromagnetic field also exhibits macroscopic quantum coherence. The most obvious example is the carrier signals for radio and TV.
{12} An electromagnetic field (EMF) it is a physical entity evoked by accelerating electric charges. The electric field is produced by stationary charges, the magnetic field by moving charges, for example by currents. The EM field can be viewed as the combination of an electric field and a magnetic field. The electromagnetic field propagates at the speed of light and interacts with charges and currents.
{13} Electromagnetic or magnetic induction is the production of an electromotive force across an electrical conductor in a changing magnetic field. Alternating electric current running through the solenoid generates a changing magnetic field. It can be shown that this field can cause, by electromagnetic induction, an electric current to flow in the adjacent wire loop.
